# Major Lower Limb Amputations: Recognizing Pitfalls

**DOI:** 10.7759/cureus.16972

**Published:** 2021-08-07

**Authors:** Lemuel Pran, Dave Harnanan, Shanta Baijoo, Andy Short, Cristo Cave, Ravi Maharaj, Shamir O Cawich, Vijay Naraynsingh

**Affiliations:** 1 Vascular Surgery, Eric Williams Medical Sciences Complex, Mount Hope, TTO; 2 Clinical Surgical Sciences, The University of the West Indies, St. Augustine, TTO; 3 Surgery, Eric Williams Medical Sciences Complex, Mount Hope, TTO; 4 Statistics, Eric Williams Medical Sciences Complex, Mount Hope, TTO; 5 Surgery, Medical Associates Hospital, St. Joseph, TTO

**Keywords:** lower extremity amputation, diabetic foot complications, major amputation, diabetic foot sepsis, multi-disciplinary team

## Abstract

Major lower extremity amputations have been an area of much concern in the Caribbean population. Hence, the purpose of this research was to investigate the current trends in major lower-extremity amputations. Data regarding all major lower-extremity amputations performed at a tertiary care institution in Trinidad and Tobago, from January 2010 to December 2016 were reviewed. The variation of yearly trends, gender, type of amputation and reason for amputation were analysed.

The yearly amputation rate demonstrated a progressive increase from 2010 to 2016, the average for the seven years was 28 per 10^5^/year. Males accounted for 59% of cases, and 60% of amputations were done above the level of the knee joint. The most common reason for amputation was control of sepsis in 71.5% of cases. A strong association between major amputations and prior intervention for a foot-related problem was observed, as 52% of the sample had a pre-existing wound or a prior minor amputation (32%). Overall, 14.5% of all amputees were able to acquire a prosthesis. Diabetes mellitus was the most consistently associated co-morbidity occurring in 91% of the study population.

Major limb amputations continue to affect our population significantly, with a rise in the amputation rate despite the introduction of a Vascular Surgical Unit. Diabetes and its foot-related complications are one of the leading causes of major lower extremity amputations. Prosthetic limb acquisition for our amputee population continues to be lacking, reflected by the low prosthetic acquisition rate observed.

## Introduction

A major lower limb amputation as defined by the Lower Extremity Amputation Study Group is the loss in the transverse anatomical plane at or proximal to the ankle joint [1,2]. The global incidence varies widely with geographic location, as well as the prevalence of diseases such as diabetes mellitus and peripheral vascular disease [2,3]. One specific group, the Native Americans, has been identified as having the highest amputation rate worldwide. This trend was observed in two tribes, the Navajo and the Chippewa Indians, documented in separate reports [2,4]. Diabetes remains a predominant problem in the Caribbean with an increasing incidence [5-7]. The International Diabetes Federation has ranked North America and the Caribbean as the number one region for the highest prevalence of diabetes (13%) in their 2019 report [8]. Additionally, international data has shown that 80% of all people with diabetes live in developing countries [8]. Consequently, a parallel incline in the rate of foot-related complications of diabetes can be anticipated. Guidelines for the prevention and management of diabetic foot infection have been provided by the International Diabetic Federation 2017. These strategies aid in the reduction of lower extremity amputations with an odds ratio of less than 2 [9]. The adequate and timely management of diabetic foot infections, therefore, cannot be overemphasized to prevent progression and further tissue loss. The most feared complication of a diabetic foot infection is undoubtedly a major amputation. Major lower limb amputations are associated with significant morbidity and mortality [10-11].

International data emerging from developed nations indicate that the leading cause of major lower limb amputations occurs as a result of peripheral arterial occlusive disease (PAOD). In the first world setting diabetes is also a major concern contributing to dysvascular disease as well as neuropathy and foot ulceration [2,12]. Environmental and genetic factors predispose the population of Trinidad and Tobago to have an increased risk for development of diabetes. Several regional studies have consistently proven that most major lower extremity amputations are done for sepsis-related complications of diabetes [13-15]. Divergence from this trend is seen in developed countries; however, a similar situation exists in the subgroups where socioeconomic status is lower with a high prevalence of diabetes [2]. The primary objective of this research was to investigate the prevalence of major lower extremity amputations and determine the impact of the introduction of a Vascular Unit on major lower extremity amputations, at our institution. Additionally, secondary objectives were to evaluate the trends observed, which predispose individuals to major lower extremity amputations and assess the mortality associated with a major lower extremity amputation.

## Materials and methods

Data collection was performed in a retrospective manner at a single tertiary care institution, in Trinidad and Tobago over a seven-year period (January 1st, 2010 to December 31st, 2016). A qualitative method was used in obtaining primary and secondary data. The study group included all patients having a major lower extremity amputation (above or below the knee) older than 18 years. A major lower extremity amputation was defined as the removal of any part of the lower limb proximal to the ankle joint. These were identified from medical records inclusive of surgeons’ and nurses’ operative logs, as well as daily operative reports. All available medical records for individual patients were reviewed by the researcher and clinical data recorded (secondary data) as specified by clinical data collection form attached. A follow-up telephone and or personal interview was conducted with these patients by the researcher.

Ethical approval was obtained from the University of the West Indies Ethics Committee (CEC079/11/15) as well as from the relevant Health Authority for conducting this project. Initially, ethical approval was obtained for the period January 2010 to December 2015, however, to increase the sample size and further power the study an application for an extension to include January to December 2016 was also approved.

The catchment population for which the tertiary care institution services was estimated to be 400,000 persons. Data was entered and analysed using SPSS 25.0 (IBM Corp., Armonk, NY) software utilizing statistical tests based on the level of data and professional query. A p-value of <0.05 was used to determine the level of statistical significance. Patients, whose medical records were not available for review, were excluded from the clinical data analysis, however parameters such as age, gender and level of amputation were included in the demographic analysis once obtained.

## Results

**Figure 1:**
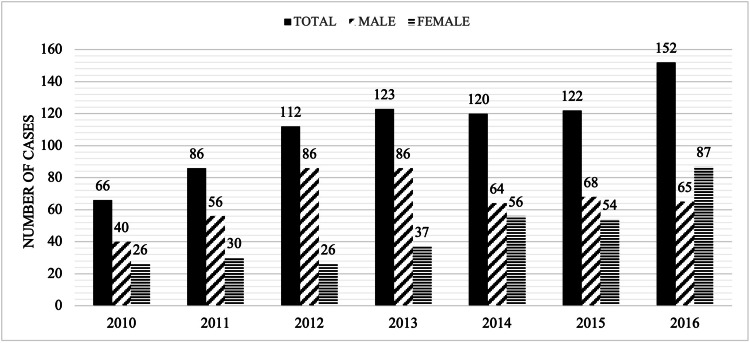
Yearly Amputation Rate for the Period 2010 to 2016

**Figure 2:**
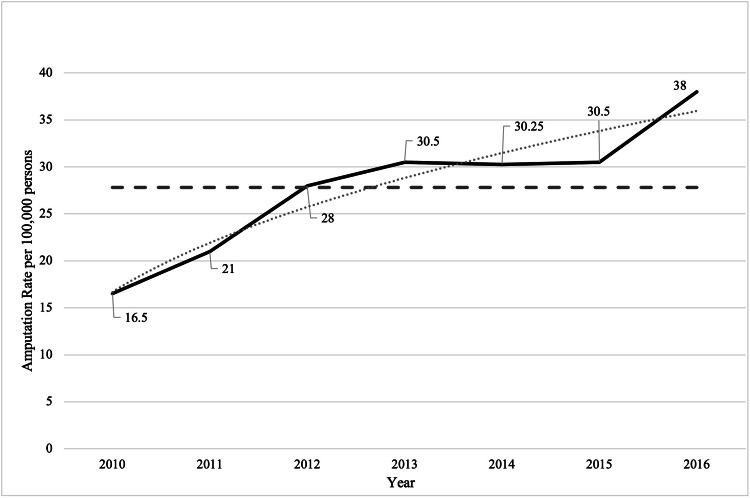
Major Lower Limb Amputation Rate per 100,000 Population for the Period 2010 to 2016

There was a total of 781 major lower limb amputations performed for seven years (Figure 1). Of these, 473 (60%) were above the knee, and 465 (59%) were males (Figure 1). A full demographic profile was obtained for 603/781 patients for 2010 to 2016, which was used for age and type of amputation analysis (Table 1). Medical reports were available for 344 cases of major lower limb amputations and these were subjected to detailed review and analysis. An upward trend has been seen for the yearly amputation rate between 2010 and 2016. There has been a two-fold increase from 66 to 152 amputations per year, as shown in Figure 1. Based on the service population of the tertiary level care institution (400,000 population), the yearly amputation rate for 2016 is 38 per 10^5^. The average for the seven years is lower at 28 per 10^5^/year, as shown in Figure 2.

**Table 1:** Age Distribution and Type of Amputation

Age Range (Years)	Above Knee Amputation (AKA) (%)	Below Knee Amputation (BKA) (%)	Total (%)
11-20	1 (0.2)	1 (0.2)	2 (0.4)
21-30	6 (1.0)	0 (0)	6 (1.0)
31-40	5 (0.8)	8 (1.3)	13 (2.1)
41-50	45 (7.5)	37 (6.1)	82 (13.6)
51-60	79 (13.1)	71 (11.8)	150 (24.9)
61-70	89 (14.8)	71 (11.8)	160 (26.6)
71-80	86 (14.3)	39 (6.5)	125 (20.8)
>80	50 (8.3)	15 (2.5)	65 (10.8)
Total	361 (59.9)	242 (40.1)	603 (100)

A significant difference was noted in the number of amputations performed on males (61.7%) compared to females (38.3 %) as well as an above-knee amputation (AKA) in both males and females (x^2^ 20.024; df 2; n 603; p <0.05). Additionally, a comparable AKA (32.8%, 198/603) and below knee amputations (BKA) (28.9%, 174/603) were performed on males, however, 27.0% (163/603) were AKA and 11.3% (68/603) were BKA performed on females (p < 0.05). The overall ratio of the above-knee to below-knee amputations was found to be 3:2.

The odds of having an amputation increased with age beyond 41 years. Overall, 80% of all amputations were performed on individuals older than 50 years with a similar trend seen for both the male and female subgroups. The 61-70-year age range accounted for most amputations (26.6%) (Table 1). In the above-knee sub-category, the highest values were observed in age groups 61-70 years (14.8%), then 71-80 years (14.3%), followed by 51-60 years (13.1%). Conversely, below knee amputations were performed mostly in the age groups 51-60/61-70 (11.8% each) followed by 71-80 (6.5%) (Table 1). The probability of difference and expected outcome observed in this population is significant (x^2^ 26.516; df 14; n 603; p 0.02). Therefore, a significant difference was seen with the number and type of amputation based on age. The overall mean age at which amputation occurred was 63.0 +/- 13.8 years, and this was comparable for both men (62.7 +/- 13.6 years) and women (63.86 +/- 14.27 years). The mean age at which a BKA was performed was 60.7 years when compared to mean age of AKA 64.7 years (ANOVA df 1; F 22.936; p <.05).

The ethnic distribution for the sample size included 39.4% Indo-Trinidadian, 36.8% Afro-Trinidadian, 18% Mixed, and 5.5% were unknown. A significant correlation was seen in patients having a major amputation and diabetes (90.4%) compared to hypertension (70.4%) (p 0.01) (Table 2 and Table 3), respectively. Additionally, diabetes was found to be more strongly associated with the Indo-Trinidadian subgroup than any other ethnicity in this sample (r 8.659; df 2; p 0.034) (Table 2). The prevalence of ischemic heart disease (IHD) was also significantly different for the Indo-Trinidadian population when compared to any other ethnic group. One-third of the Indo-Trinidadian sample population (45/136) suffered from cardiac disease, accounting for approximately 55% (45/82) of all patients with a history of ischemic heart disease (r 11.781; df 3; p .008) (Table 4). There were no significant differences regarding the prevalence of hypertension and end-stage renal disease for any ethnic group.

**Table 2:** Prevalence of Diabetes Mellitus Across the Various Ethnic Groups

			Ethnicity				Total
			Indo-Trinidadian	Afro-Trinidadian	Mixed	Unknown	
Diabetes Mellitus	No	Count	5	16	9	2	32
		% within Diabetes Mellitus	15.6%	50.0%	28.1%	6.3%	100.0%
		% within Ethnicity	3.7%	12.6%	14.5%	10.5%	9.3%
	Yes	Count	131	111	53	17	312
		% within Diabetes Mellitus	42.0%	35.6%	17.0%	5.4%	100.0%
		% within Ethnicity	96.3%	87.4%	85.5%	89.5%	90.7%
Total		Count	136	127	62	19	344
		% within Diabetes Mellitus	39.5%	36.9%	18.0%	5.5%	100.0%
		% within Ethnicity	100.0%	100.0%	100.0%	100.0%	100.0%

**Table 3:** Prevalence of Hypertension Across the Various Ethnic Groups

			Ethnicity				Total
			Indo-Trinidadian	Afro-Trinidadian	Mixed	Unknown	
Hypertension	No	Count	37	38	20	5	100
		% within Hypertension	37.0%	38.0%	20.0%	5.0%	100.0%
		% within Ethnicity	27.2%	29.9%	32.3%	26.3%	29.1%
	Yes	Count	99	89	42	14	244
		% within Hypertension	40.6%	36.5%	17.2%	5.7%	100.0%
		% within Ethnicity	72.8%	70.1%	67.7%	73.7%	70.9%
Total		Count	136	127	62	19	344
		% within Hypertension	39.5%	36.9%	18.0%	5.5%	100.0%
		% within Ethnicity	100.0%	100.0%	100.0%	100.0%	100.0%

**Table 4:** Prevalence of Ischemic Heart Disease Across the Various Ethnic Groups

			Ethnicity				Total
			Indo-Trinidadian	Afro-Trinidadian	Mixed	Unknown	
Ischemic Heart Disease	No	Count	91	102	54	15	262
		% within Ischemic Heart Disease	34.7%	38.9%	20.6%	5.7%	100.0%
		% within Ethnicity	66.9%	80.3%	87.1%	78.9%	76.2%
	Yes	Count	45	25	8	4	82
		% within Ischemic Heart Disease	54.9%	30.5%	9.8%	4.9%	100.0%
		% within Ethnicity	33.1%	19.7%	12.9%	21.1%	23.8%
Total		Count	136	127	62	19	344
		% within Ischemic Heart Disease	39.5%	36.9%	18.0%	5.5%	100.0%
		% within Ethnicity	100.0%	100.0%	100.0%	100.0%	100.0%

A previous minor amputation occurred in 110 of 344 patients and was found to have an increased likelihood of having a major amputation (p <0.05) (Table 5). A patient with an open wound was found to be a strong predictor preceding a major amputation; this was evident as 51.8% of the population was found to have an ulcer on the affected leg (p 0.006) (Table 5).

**Table 5:** Type of Amputation and Association Between Prior Minor Amputation and Mechanism of Injury

		Type of Amputation		Total		
		Above Knee	Below Knee		Likelihood Ratio	p-Value
Prior Minor Amputation	No	149	85	234		
	Yes	46	65	110	16.112	<0.05
Total		195	150	344		
Mechanism of Injury						
Unknown		64	41	104		
Penetrating Injury		13	17	30		
Minor Foot Trauma		4	10	14		
Open Wound		99	80	179	15.657	.006
Acute Ischemia		15	2	17		
Total		195	150	344		

The average length of stay for the entire sample was 15.2 days. Patients who had above-knee amputation had a mean length of stay of 14.0 days (N 178; SD 12.006; SE 0.900) as compared to below-knee amputees of 16.7 days (N 141; SD 13.321; SE 1.122). Despite an approximate three-day prolonged stay for the below-knee amputation group, this difference was not statistically significant (t 1.912; df 317; p 0.057). An inverse correlation between age and length of stay was present; however, this relation also failed to reach statistical significance (r .108; N 319; p 0.054).

**Figure 3:**
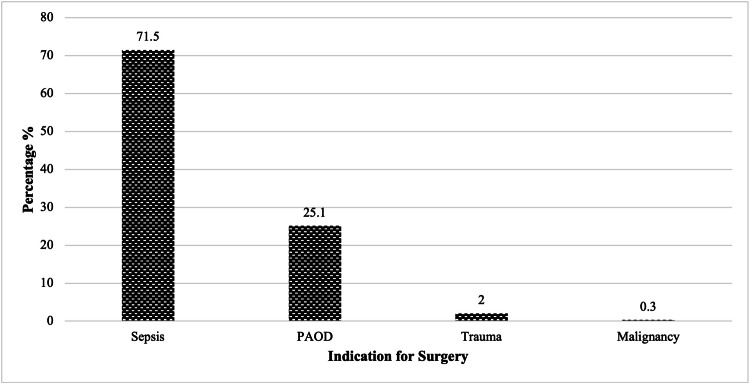
Indication for Major Lower Limb Amputation

Many patients who had an amputation presented more than seven days after the onset of symptoms accounting for 79% of cases (272/344). Sepsis was the most common indication for amputation occurring in 71.5% of the sample, as shown in Figure 3. Primary amputation was performed in 91% of cases (313/344), with the average secondary amputation rate of 12% for the years 2015 and 2016 (the period for which Vascular Unit provided revascularization).

There were several factors which were found to be positively associated with ambulation status and prosthesis use. Persons who had a below-knee amputation were more likely to ambulate with and without a prosthesis compared to above-knee amputees (p <0.05). Additionally, preoperative higher Eastern Cooperative Oncology Group (ECOG) status was associated with higher rates of ambulation with and without prosthesis. Among individuals who attained ambulation with a prosthesis, 85% were ECOG 0 and 1 preoperative status (p 0.004). Overall, 14.5% of the sample population attained a prosthetic limb.

## Discussion

Major lower extremity amputations are generally considered a last resort and are usually done as a salvage procedure. Diabetes is known to predispose an individual to develop severe foot infections, which if inadequately treated, can lead to a major amputation. In Trinidad and Tobago, the prevalence of diabetes is estimated at 11.7% to 12.5% according to the International Diabetes Federation and World Health Organisation, respectively [8,16].

There are several pathways for which foot ulceration, in a person with diabetes, can occur as highlighted by Boulton [17]. However, the natural sequelae of diabetic foot infection in the developing country differs from the first world setting. Poor glycaemic control, lack of patient education and limited primary care access are all factors which create a challenge in the management of this condition [14]. Sensory neuropathy is a predominant feature leading to loss of proprioception and sensation. The slipping slipper sign (SSS) was found to be a marker of severe peripheral neuropathy in a Trinidad-based study, while in patients with active foot sepsis the SSS was prevalent in 65% of cases [18]. Therefore, it is not surprising that the most common mechanism resulting in a diabetic foot infection is minor traumatic injury [19]. Diabetes-related foot infections are the number one cause of major lower extremity amputations in the developing world [20]. This project has highlighted that 91% (313/344) of the sample population were in fact, people with diabetes. Furthermore, diabetes-related foot sepsis was found to be the number one underlying aetiological factor in 69% (236/344) p-value <0.05. These findings are in keeping with data published by Naraynsingh et al. in 2002 [14]. In a further study published by Solomon et al. in 2008, the sample included a significant portion of diabetic individuals who underwent a major amputation [13]. Regional studies have also provided supporting evidence concerning the magnitude of diabetes prevalence and its related complications resulting in lower extremity amputations. Hennis et al. reported 96.4% of amputees having diabetes, and later Sumpio et al. documented an 85% rate, both studies were based on a Barbados population [20,21].

The overall amputation rate for the seven years was 28 per 10^5^/year, ranging from 16.5 to 38 per 10^5^/year. The amputation rate of 16.5 per 10^5^ for 2010 reflected a comparable value to 2005 of 13.85 per 10^5^/year previously documented [13]. There are several variables to be considered to understand the reasons for an increasing amputation rate of 38 per 10^5^ in 2016. Since the St. Vincent Declaration for the Caribbean, an aggressive approach to diabetic foot complications and major amputations was adopted. This initiative was proposed in 1989, and the intent was to reduce diabetes-related complications. An area where targeted improvement was required was the major amputation rate. It was proposed through various programs to reduce the major amputation rate by 50% in five years [22]. It was expected that with the introduction of a Vascular Unit capable of both endovascular and open interventions, there would have been a significant decrease in the amputation rate. Contrary to the St. Vincent Declaration initiative, if the last five years of this study is evaluated, a 35% increase in the major amputation rate is observed from 2010 to 2016.

In the United States diabetic population, an amputation follows a foot ulcer in 80% of cases [17]. However, in our population, minor foot traumatic injury is the precipitating factor, documented by Islam et al. [19]. A bimodal distribution of major amputations can be observed, with the first peak on the index admission where sepsis is overwhelming, and limb salvage is not possible. Primary care can target the first peak in which patients admitted require a major amputation as a life-saving procedure. Aggressive programmes to address patient education on foot care, management of co-morbid conditions with best medical therapy, encourage patients to become self-aware and take an active role in their health care should be foremost [23]. Additionally, foot screening programmes will identify individuals with an at-risk foot, who can then be enrolled in an appropriate surveillance program. Furthermore, in the event, the patient develops some form of foot injury they are aware and will be equipped to access treatment through a systematic patient care pathway. Aside from decreasing the number of amputations done on the first admission, this strategy decreases the number of class 4 diabetic foot procedures that are performed and so reduces the number of surgically created wounds [24,25].

In cases where limb salvage is intended, surgical debridement is instituted for infection control which results in a varying degree of tissue loss. These surgically created wounds are then left to heal by secondary intention, which may take several weeks to months in some cases. During this period of wound healing, there is another surge in the number of patients requiring major amputations due to reinfection of a non-healing or delayed healing wound. Pre-existing wounds were present in approximately 52% of individuals, which conferred a statistically significant risk for major amputation in this population (p 0.006). This highlights a major failure to adequately provide aggressive surveillance for these patients. Any diabetic patient who required admission for a foot infection automatically becomes a high risk for major amputation. In a locally conducted prospective study by Islam et al., the major amputation rate was found to be 14% for patients admitted for diabetic foot problems [19]. Hence, on discharge enrolment into a foot surveillance program is essential to ensure wound healing promptly and evaluate the progression of the wound. The presence of a deep ulcer or a positive probe to bone test was found to be independent risk factor for any type of lower limb amputation in a study done in conjunction with the International Working Group on the Diabetic Foot (IWGDF) [26].

Immediate amputation due to limb or life-threatening sepsis is predominant in developing countries. In this review, we note that a significant portion of amputations was done as primary procedures, with no input from the Vascular Unit. Furthermore, the primary amputation rate before and after the introduction of the Vascular Surgery Unit was both comparable. Another variable which should be taken into account is the fact that more than 75% of patients included in the study presented more than seven days after the onset of symptoms. Hence this raises the issue of appropriate, timely patient referral and dedicated service for the management of diabetic foot infection, in addition to wound care.

Another challenge when setting up a novel service, is the tendency to fall into the “Sole Practitioner Syndrome”, highlighted by Cawich et al. [27]. However, to successfully lower the major amputation rate, significant inputs from Vascular Scientists, Specialist Vascular Nurses, Podiatrists, Diabetologist, Specialist Wound Care Nurses in addition to the Vascular Surgeon are all mandatory. A multidisciplinary management approach has a definitive benefit and improved outcomes. There were several observational studies done which assessed the effect of designated foot and wound clinics on the major lower limb amputation rate. In a 16-year Danish study by Rasmussen et al., there was a reduction in below ankle and below-knee amputation rate by approximately 10% and 15%, respectively [28]. Although human resources and expertise play a vital role in the provision of a service, many other supportive facilities are also as equally important. These include dedicated operating theatre (open and endovascular) facilities, consumables, a dedicated diabetic foot clinic, vascular laboratory, community and satellite wound care clinics. These factors further emphasize the need for foot care programs with a similar approach in the local setting.

Traditionally, mortality rate post major lower limb amputation is high, which may range 30% in the first 30-day and overall rates of 44% and 77% at one and five years, respectively [10,11]. The in-hospital mortality rate was 10%, with a 30-day mortality of 15% and an overall mortality rate of 34% at two years. This trend is observed for several reasons. Amputations are usually done as an emergent or urgent surgical procedure according to the Confidential Enquiry into Perioperative Death (CEPOD) classification [29]. These patients usually have significant co-morbid conditions such as diabetes mellitus and hypertension. In our sample population, 90% were diabetics, 70% were hypertensive, 24% were diagnosed with IHD, and 15% suffered from end-stage renal disease. Additionally, due to their medical history, a significant number of these patients may have undiagnosed cardiovascular disease. There is very little time to fully optimize these individuals in preparation for a major surgical procedure. Furthermore, the most common indication for surgery was sepsis occurring in 71.5% of cases, which when compounded with the physiological demands of a major operation significantly increases the risk of an acute cardiac event intra- and postoperatively. The mortality rate is considered to be relatively low for the population defined. Various factors can account for this, mainly because data collection was performed retrospectively. The effect of convenience bias should be considered as medical records for patients who died in hospital were more difficult to retrieve. Additionally, patients who were not contactable either personally in the surgical outpatient clinic or via telephone were likely to be of lower health and performance status.

Although this research has provided further insight into the problem of lower extremity amputations in our population, it is not without limitations. The study was a pilot, single-institution project, and so the data obtained is reflective for this catchment population. Despite obtaining a sample size with an acceptable confidence interval and margin of error, a significant portion of the target population was not included. Additionally, some patients were lost to follow up, unfortunately, and a high degree of convenience bias was present. These are issues encountered with retrospective data collection, and so a prospective approach will be the ideal in the future. The intent is to extend this research to all major hospitals to obtain a more extensive and broader sample size reflective of population-based data.

## Conclusions

The major lower limb amputation rate had increased from 66 per year in 2010 to 152 per year in 2016. This rise has been noted to be multifactorial, inclusive of increasing population with poorly controlled co-morbidity, preferential referral pattern and the challenge of patients with late-stage disease. Strategies which may dampen this trend include the provision of adequate health care at the primary level, development of foot clinics and extensive patient education. A multidisciplinary team with adequate support services is essential for lowering of the major lower limb amputation rate.
